# Spinal Cord Injury—Assessing Tolerability and Use of Combined Rehabilitation and NeuroAiD (SATURN Study): Protocol of An Exploratory Study In Assessing the Safety and Efficacy of NeuroAiD Amongst People Who Sustain Severe Spinal Cord Injury

**DOI:** 10.2196/resprot.6275

**Published:** 2016-12-05

**Authors:** Ramesh Kumar, Ohnmar Htwe, Azmi Baharudin, Mohammad Hisam Ariffin, Shaharuddin Abdul Rhani, Kamalnizat Ibrahim, Aishah Rustam, Robert Gan

**Affiliations:** ^1^ Department of Neurosurgery Faculty of Medicine University Kebangsaan Malaysia Medical Centre Kuala Lumpur Malaysia; ^2^ Department of Orthopaedics and Traumatology Faculty of Medicine University Kebangsaan Malaysia Medical Centre Kuala Lumpur Malaysia; ^3^ Medical Affairs Moleac Biopolis Way Singapore

**Keywords:** spinal cord injury, NeuroAiD, MLC601, MLC901, safety, recovery, efficacy

## Abstract

**Background:**

Spinal cord injury (SCI) is a devastating condition with limited therapeutic options despite decades of research. Current treatment options include use of steroids, surgery, and rehabilitation. Nevertheless, many patients with SCI remain disabled. MLC601 (NeuroAiD), a combination of natural products, has been shown to be safe and to aid neurological recovery after brain injuries and may have a potential role in improving recovery after SCI.

**Objective:**

The aim of this study is to evaluate the safety and efficacy of NeuroAiD amongst people who sustain SCI in the study setting.

**Methods:**

Spinal Cord Injury—Assessing Tolerability and Use of Combined Rehabilitation and NeuroAiD (SATURN) is a prospective cohort study of patients with moderately severe to severe SCI, defined as American Spinal Injury Association (ASIA) Impairment Scale (AIS) A and B. These patients will be treated with open-label NeuroAiD for 6 months in addition to standard care and followed for 24 months. Anonymized data will be prospectively collected at baseline and months 1, 3, 6, 12, 18, and 24 and will include information on demographics; main diagnostics; and neurological and functional state assessed by the Spinal Cord Independence Measure, ASIA—International Standard for Neurological Classification Spinal Cord Injury, and Short Form (SF-8) Health Survey. In addition, NeuroAiD treatment, compliance, concomitant therapies, and side effects, if any, will be collected. Investigators will use a secured online system for data entry. The study is approved by the ethics committee of Hospital University Kebangsaan Malaysia.

**Results:**

The coprimary endpoints are safety, AIS grade, and improvement in ASIA motor score at 6 months. Secondary endpoints are AIS grade, ASIA motor scores and sensory scores, Spinal Cord Independence Measure (SCIM), SF-8 Health Survey, and compliance at other time points.

**Conclusions:**

SATURN investigates the promising role of NeuroAiD in SCI especially given its excellent safety profile. We described here the protocol and online data collection tool we will use for this prospective cohort study. The selection of moderately severe to severe SCI provides an opportunity to investigate the role of NeuroAiD in addition to standard rehabilitation in patients with poor prognosis. The results will provide important information on the feasibility of conducting larger controlled trials to improve long-term outcome of patients with SCI.

**Trial Registration:**

Clinicaltrials.gov NCT02537899; https://clinicaltrials.gov/ct2/show/NCT02537899 (Archived by WebCite at http://www.webcitation.org/6m2pncVTG)

## Introduction

### Background

Spinal cord injury (SCI) is a devastating neurological disorder that affects thousands of individuals each year. Global incidence rate of traumatic SCI in 2007 was estimated at 23 cases per million (133,000 to 226,000 cases per annum) and prevalence was between 236 to 4187 per million [1].

Over the past decades, much progress has been made in our understanding of the molecular and cellular events in SCI, providing insights into important mechanisms of tissue damage and failure of regeneration of injured neurons. Current treatment options for SCI include the use of high-dose methylprednisolone and surgical interventions to stabilize and decompress the spinal cord in the acute period, while rehabilitation is provided as long-term management. There is currently no treatment that enhances recovery after the injury, and SCI remains to be a devastating condition for which therapeutic options are still limited [2].

Three decades of clinical research on interventions to improve neurological outcomes in persons with SCI has not translated the promise of preclinical discovery into a consensus standard of care treatment. Nonetheless, SCI researchers remain hopeful that advances in preclinical discovery coupled with improved clinical trial performance will yield effective restorative treatment [3].

There have been many lessons learned from past failures in clinical trials, including patient selection based on knowledge of prognosis for spontaneous natural recovery and other eligibility criteria, clinical trial design, and outcome measures [4-7]. By taking the lessons into consideration in selecting a series of cases to be treated, important insights may be gained into the potential role of NeuroAiD as a therapy for SCI.

MLC601 (NeuroAiD, Nu-rAiD) is a combination of 14 natural ingredients indicated as treatment for poststroke recovery widely used in China and in many countries in Asia [8,9]. In Europe, a simplified formulation of the product, MLC901 (NeuroAid II, NurAiD II), consisting of 9 herbal components is available and will soon be available in Asian countries as well. Both formulations shall collectively be referred to as “NeuroAiD” in this study.

NeuroAiD efficacy and safety are supported by preclinical and clinical studies. The neuroprotective and neuroproliferative properties of NeuroAiD have been extensively elucidated during in vitro and in vivo experiments using animal and cellular models of focal and global ischemia [8-11]. In addition, research on its positive effects in traumatic brain injury (TBI) has recently been published [12,13]. What are remarkable are the effects of NeuroAiD on neurogenesis and neurorestoration beyond mere neuroprotection.

Case series reports of the use of NeuroAiD in neurosurgical conditions have been the subject of publications and presentations in international neurosurgical congresses [14-16]. In addition, there are several ongoing studies on the use of NeuroAiD in poststroke cognitive impairment and TBI [17-19].

The clinical data on NeuroAiD, however, are most well-reported in stroke. A systematic review of randomized clinical trials on NeuroAiD showed its benefits in improving functional outcomes and neurological deficits with 3 months treatment among patients with ischemic stroke in the preceding 1 week to 6 months [20-25]. Subsequently, NeuroAiD was investigated in acute ischemic stroke within 72 hours of onset which demonstrated the treatment effect to be larger in postacute and relatively more severe stroke at 3 months [26-29]. The updated meta-analysis showed a pooled odds ratio in favor of NeuroAiD [27]. Furthermore, treatment with NeuroAiD was associated with a reduction in risk of early vascular events after a stroke [30]. More recently, the extension study of this randomized, placebo-controlled, double blind trial has provided evidence of its benefit on long-term functional outcome persisting over time up to 18 months after a stroke with an excellent safety profile [31].

Since 2001 when it was marketed in China, there have been minimal serious side effects reported to date with the use of NeuroAiD. The common side effects reported from NeuroAiD were mostly mild and transient. Excellent clinical safety has been demonstrated in published clinical trials which reported the more common adverse events being gastrointestinal (nausea, vomiting, discomfort, diarrhea, dry mouth) and headache [20-34]. Safety studies in humans have shown that NeuroAiD, given alone or combined with aspirin, had no effect on clotting and coagulation [35]. Furthermore, there was no effect on hematological, hemostatic, and biochemical parameters or electrocardiogram in normal and stroke patients, even when started within 48 hours of stroke onset [35-37].

### Study Objectives

The primary objective of this cases series is to evaluate the safety and potential role of NeuroAiD in SCI.

## Methods

### Study Design and Subject Eligibility

**Figure 1:**
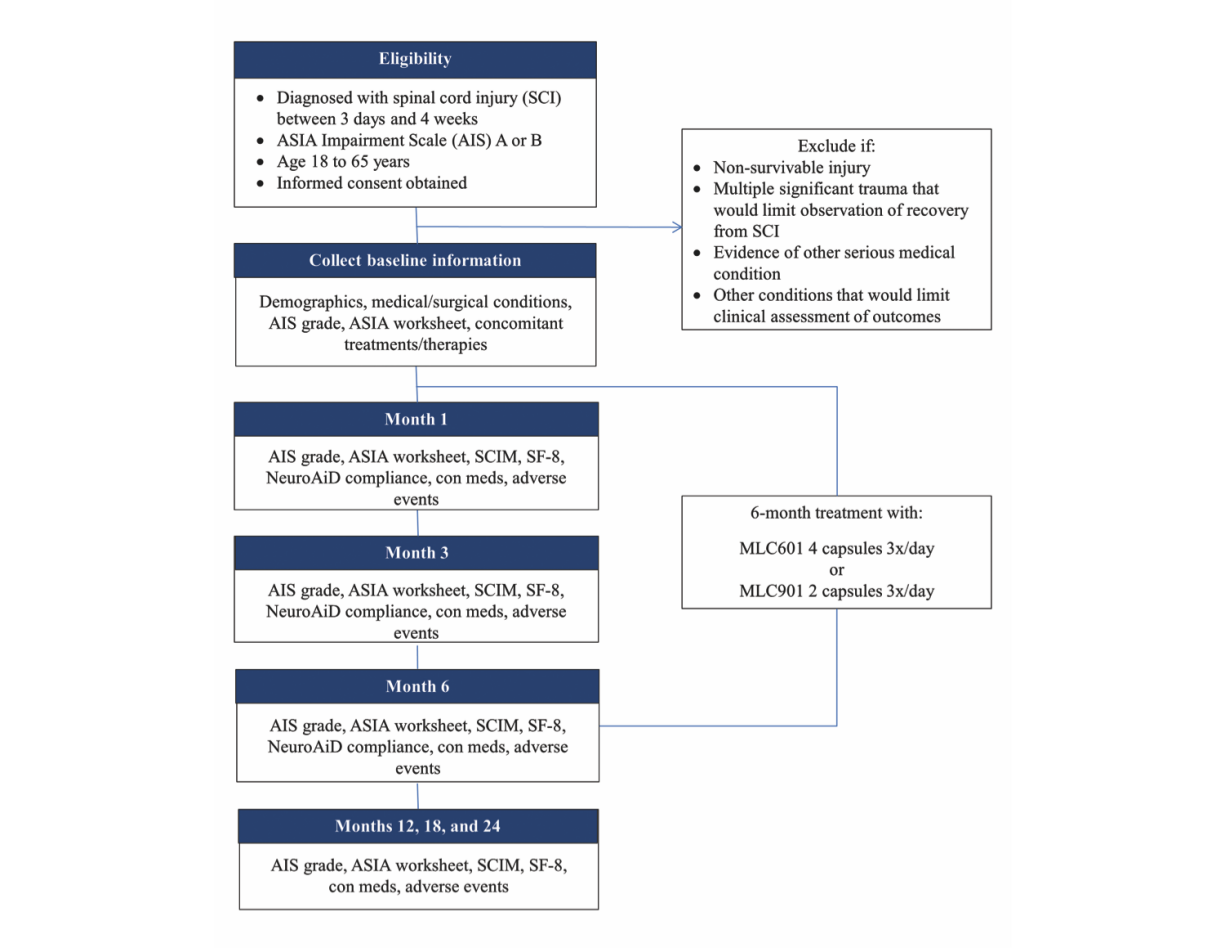
Spinal Cord Injury—Assessing Tolerability and Use of Combined Rehabilitation and NeuroAiD (SATURN) study flowchart.

Spinal Cord Injury—Assessing Tolerability and Use of Combined Rehabilitation and NeuroAiD (SATURN) is a prospective cohort study of patients with moderately severe to severe SCI treated with open-label NeuroAiD in addition to standard care (Figure 1).

As this is an open-label study, inclusion of patients with SCI who are likely to spontaneously recover may confound the results of the study. Potential prognostic factors are severity of SCI and time from injury to assessment [4]. Therefore, patients are included in the study if they meet all of the following inclusion and none of the exclusion criteria (see Textbox 1).

**Selection criteria for the study.**

Inclusion criteria:

- Male or female
- Age 18 to 65 years
- Diagnosed with SCI between 3 days and 4 weeks
- American Spinal Injury Association (ASIA) Impairment Scale (AIS) A or B
- Informed consent obtained

Exclusion criteria:

- Nonsurvivable injury
- Multiple significant trauma (ie, significant intracranial and extracranial injuries including limb fractures) that would limit observation of recovery from spinal cord injury
- Other conditions that would limit clinical assessment of outcomes (eg, dementia, demyelinating disease, autoimmune disease)
- Refusal of treatment or contraindication to NeuroAiD

### Study Setting and Recruitment of Participants

Information about the study will be disseminated to the hospital and department staff. Any potential subject referred to the study team will be prescreened for potential eligibility. Permission to approach the patient or their legal representative will be obtained from the primary physician. Participants who fulfil the eligibility criteria will be recruited while still in the hospital for treatment or rehabilitation within the specified time window from injury. Informed consent will be obtained from all participants after discussion of the nature, purpose, and potential risks of the study.

### Treatment

Each 400 mg capsule of MLC601 contains 9 herbal ingredients (extracts of *Radix astragali*, *Radix salviae miltiorrhizae*, *Radix paeoniae rubra*, *Rhizoma chuanxiong*, *Radix angelicae sinensis*, *Carthamus tinctorius*, *Prunus persica*, *Radix polygalae*, and *Rhizoma acori tatarinowii*) and 5 nonherbal components (Hirudo, *Eupolyphaga seu steleophaga*, *Calculus bovisartifactus*, *Buthus martensii*, and *Cornu saigae tataricae*). MLC901 contains only the 9 herbal extracts.

The product is available in capsule form and administered orally or the contents may be diluted in water and administrated via gastric tube. The dosage is 4 capsules 3 times a day for MLC601 and 2 capsules 3 times a day for MLC901. The treatment duration is 6 months.

The capsules should be kept sealed until opened for administration and stored below 30°C in a dry place. NeuroAiD is manufactured according to applicable control measures that ensure the consistency and quality of the product from batch to batch and adhere to good manufacturing practice. The active ingredients and finished product are subjected to full quality control testing for safety.

All participants are allowed to receive standard care and other therapies and treatments including, but not limited to, surgery, rehabilitation, and other types of care deemed appropriate and as prescribed by their physician. There is no restriction to the use of any other treatment as recommended by the treating physician although other treatments should be recorded in the database.

### Variables Collected

Subjects will undergo assessments at baseline and at months 1 (±7 days), 3 (±14 days), 6 (±14 days), 12 (±30 days), 18 (±30 days), and 24 (±30 days) (see Table 1). Information collected is meant to specifically address the objectives of the study.

**Table 1:** Schedule of Spinal Cord Injury—Assessing Tolerability and Use of Combined Rehabilitation and NeuroAiD (SATURN) study procedures.

Information collected	Base-line	Month 1 ±7d	Month 3 ±14d	Month 6 ±14d	Month 12 ±30d	Month 18 ±30d	Month 24 ±30d
Demographics	X						
Diagnosis and medical condition	X						
Surgical history	X						
AIS^a^ grade	X	X	X	X	X	X	X
ASIA ISNCSCI^b^ worksheet	X	X	X	X	X	X	X
NeuroAiD treatment/compliance	X	X	X	X			
SCIM^c^		X	X	X	X	X	X
SF-8^d^ Health Survey	X	X	X	X	X	X	X
Concomitant treatments/therapies	X	X	X	X	X	X	X
Adverse events	X	X	X	X	X	X	X

^a^AIS: American Spinal Injury Association Impairment Scale.

^b^ASIA ISNCSCI: American Spinal Injury Association International Standard for Neurological Classification Spinal Cord Injury.

^c^SCIM: Spinal Cord Independence Measure.

^d^SF: Short Form.

Data collected at baseline immediately prior to or at the start of NeuroAiD treatment will include

- Demographics data: date of birth, gender, ethnicity
- Details of diagnosis of SCI: date of occurrence; spinal cord level; cause of spinal cord injury; magnetic resonance imaging, computed tomography, or x-ray results; presence of specific sequelae of SCI (eg, respiratory failure, pneumonia, circulatory problems, spasticity and muscle tone, autonomic dysreflexia, pain, bladder and bowel dysfunctions, sexual dysfunction)
- AIS grade (pre- and postsurgery if patient undergoes surgery)
- ASIA International Standard for Neurological Classification Spinal Cord Injury (ISNCSCI) worksheet (pre- and postsurgery if patient undergoes surgery)
  - Motor subscores for each limb and total motor score based on ASIA
  - Sensory subscores based on ASIA
  - Other items in the ASIA ISNCSCI worksheet
- NeuroAiD use: date started and dose
- Concomitant medications and treatments including any surgical intervention (date performed), treatments administered (start date and stop date), and rehabilitation (ie, physical and occupational therapies [start date, stop date, location—rehabilitation center, home-based]).

Data collected from month 1 to month 24 will include compliance with intake of NeuroAiD, occurrence of any adverse event, Spinal Cord Independence Measure (SCIM), and Short Form (SF)-8 Health Survey, in addition to the other clinical assessments performed at baseline.

### Data Collection

The investigators or designated personnel must record all required participant data in their entirety to ensure accurate interpretation of data. An explanation must be documented for any missing data. Data will be collected through an online data entry system [38] which is compliant with the Health Information Privacy and Security Act. Contributors to the study will be provided secured access accounts with username and password. Paper report forms are available if online submission is not possible (eg, Internet downtime, computer malfunction, power outage) but online entry is the preferred mode of data collection. If paper forms are used, data must be written in a neat and legible manner using black or blue ballpoint pen to ensure the clarity of the reproduced copy of all completed forms which are signed and dated. The completed online or paper forms shall serve as the source documents. No other medical record or source document will be required for this study.

### Study Endpoints

The primary endpoints for this study are the AIS grade at 6 months, the improvement in ASIA total motor score at 6 months compared to baseline, and safety.

The secondary endpoint measures will be the neurological recovery of the subjects as assessed by

- AIS grade at 1, 3, 12, 18, and 24 months
- ASIA motor scores at 1, 3, 12, 18, and 24 months
- ASIA sensory scores at 1, 3, 6, 12, 18, and 24 months
- SCIM at 1, 3, 6, 12, 18, and 24 months
- SF-8 Health Survey at 1, 3, 6, 12, 18, and 24 months
- Compliance to NeuroAiD at 1, 3, and 6 months

### Safety Considerations

#### Definition of Adverse Event and Serious Adverse Event

An adverse event is defined as any untoward medical occurrence in a person administered a product which does not necessarily have a causal relationship with this treatment. An adverse event is considered a serious adverse event if it results in death, persistent or significant disability, abortion, congenital anomaly, or birth defect; is life-threatening; or requires inpatient hospitalization or prolongation of existing hospitalization.

#### Side Effect (or Adverse Drug Reaction)

A side effect is an effect, whether therapeutic or adverse, that is secondary to the one intended. It can also apply to beneficial, but unintended, consequences of the use of a treatment. For the purpose of this study, a side effect (or adverse drug reaction) is any unintended adverse event that is related to the use of the treatment, NeuroAiD. Based on causality as defined by the World Health Organization–Uppsala Monitoring System [39], any adverse event that is considered by the treating physician as being possibly, probably, or definitely related to NeuroAiD would be considered as a side effect.

#### Reporting of Adverse Events and Side Effects

All adverse events or laboratory abnormalities that develop during the course of the participant’s treatment will have to be reported in the study. Adverse events should be reported as a diagnosis or syndrome. If this is not possible, the specific symptom or abnormality may be entered. The start date of first onset of any sign or symptom of the event and stop date (date the event is considered to have resolved, if resolved) should be entered. Seriousness should be classified according to the definition of a serious adverse event.

Side effects that are reported in the study, especially those categorized as serious, will trigger a request for more medical information to ascertain details and need for reporting as serious adverse reaction to local regulatory authorities, if required.

#### Follow-Up of Adverse Events

Any adverse event must be followed until resolution, the condition stabilizes, the event is explained, or the participant is lost to follow-up. The physician is responsible to ensure that standard medical diagnostic or therapeutic management, if any, is performed.

#### Common Side Effects Reported From NeuroAiD

The common side effects reported from NeuroAiD use were mostly mild and transient. These include dry mouth, nausea, vomiting, abdominal discomfort, diarrhea, and headache. In many cases, the side effect resolved with reduction of the dose by half for one week, resumed at the usual dose after resolution of the symptom.

### Ethical Considerations

Approval from the ethics committee of the Hospital University Kebangsaan Malaysia has been obtained for this study. Patient information sheet and informed consent form as approved by the ethics committee will be used to explain to the subjects the nature and purpose of their participation prior to performing any study-related procedures.

The potential risks involved in participating in the study will be explained to the patient. The medical judgement regarding the use of NeuroAiD as treatment for SCI is a joint decision of the physician and the patient or legal representative. This decision must be arrived at prior to and without consideration of the patient’s potential participation in the study registry. Possible common side effects of the treatment and in very rare cases severe allergic reaction or unexpected life-threatening events will be explained.

### Statistical Consideration

The target number of participants for this exploratory cohort study is set at 30. Descriptive statistics will be used to summarize data. Outcome assessments will be compared to baseline and previous observations. Comparison with appropriate historical controls with the same AIS grading and timeframe will be performed [4]. Multiple variable analyses will be used to identify predictors of better outcome, when appropriate. Other analyses deemed appropriate by the investigators and statistical consultant will also be performed.

### Study Administration and Oversight

The study shall be carried out in the Hospital University Kebangsaan Malaysia, Kuala Lumpur, Malaysia, led by the principal investigator (RK) and assisted by coinvestigators (OH, AB, MHA, SAR, KI).

### Data Management

The online data entry system is compliant with the Health Information Privacy and Security Act. Data will be maintained in a secured database in Moleac (Singapore) accessible only to relevant personnel. Subjects’ identities will be recorded only as initials with identification numbers. Specific individual information in the study will not be shared with other persons, entities, or companies unless required by legal authorities. Collective anonymized information will be summarized and reviewed. These summaries may be presented to stakeholders (eg, physicians, regulatory authorities, attendees in a conference) and/or published in scientific journals.

### Adherence and Amendments to Protocol

Study investigators must adhere to the protocol and ensure that it is strictly followed. If necessary, an amendment may be implemented only after approval is obtained for the amendment from the ethics committee, except where necessary to eliminate an immediate hazard to participants or when changes involve any logistical or administrative aspects of the study (eg, change of personnel, change of telephone number). Amendments may not remove any of the basic data elements as described in this protocol.

### Closure of Study

The study shall be closed when the number of study participants has been reached and the investigators agree that further inclusion of participants is no longer relevant or necessary. The closure will occur in steps, starting with informing investigators of the plan to discontinue inclusion of more participants and until after the follow-up of the last participant has been completed. The database will subsequently be locked upon an agreed timeframe with the principal investigator.

## Results

The study is currently recruiting patients and is expected to complete in June 2018.

## Discussion

The treatment options for SCI are limited. The reasons for the failure of earlier clinical trials to find new therapies in SCI have been extensively discussed [3-7]. Exploring new therapeutic strategies from natural substances is attractive and has gained much attention recently. The SATURN study investigates the promising role of NeuroAiD in SCI using an open-label cohort study design which is appropriate for a first-ever safety and efficacy study of a new treatment in this condition. Nevertheless, the design of the study takes much of past learnings into consideration. SATURN would include more severe and less acute patients among whom true treatment effect may be better demonstrated by reducing spontaneous recovery as a confounder. Neurological, functional, and quality of life outcomes are measured. As recovery and neuroplasticity may take time to accrue, the period of treatment and observation is extended to detect any delayed benefits which may not be apparent during the first few months of SCI. Published cohorts of patients with the same severity and timeframe may serve as historical controls. The knowledge gained from SATURN will certainly provide important insights on safety and efficacy in planning future clinical trials.

## Abbreviations

AIS: American Spinal Injury Association Impairment Scale
ASIA: American Spinal Injury Association
ISNCSCI: International Standard for Neurological Classification Spinal Cord Injury
SATURN: Spinal Cord Injury—Assessing Tolerability and Use of Combined Rehabilitation and NeuroAiD
SCI: spinal cord injury
SCIM: Spinal Cord Independence Measure
SF-8: Short Form 8-Item Health Survey
TBI: traumatic brain injury
